# Impact of COVID-19 lockdown on physical activity behaviours of older adults who participated in a community-based exercise program prior to the lockdown

**DOI:** 10.1371/journal.pgph.0001217

**Published:** 2022-11-11

**Authors:** Kaoru Nosaka, Caitlin Fox-Harding, Kazunori Nosaka

**Affiliations:** 1 School of Medical and Health Sciences, Edith Cowan University, Joondalup, Western Australia, Australia; 2 School of Nursing and Midwifery, Edith Cowan University, Joondalup, Western Australia, Australia; 3 Exercise Medicine Research Institute, Edith Cowan University, Joondalup, Western Australia, Australia; Babcock University, NIGERIA

## Abstract

This study investigated the impact of the 2020 COVID-19 lockdown on community-dwelling older adults attending a community-based exercise program to seek strategies to keep them active during self-isolated situations. A two-phase mixed methods approach included a survey followed by in-person focus groups. Forty-eight participants, with 32 starting a community-based exercise program before the lockdown and 16 joining the program after the lockdown, completed a questionnaire survey about physical activities before and during the lockdown. This was followed by three focus groups (26 participants in total) to identify factors influencing physical activity behaviours found in the survey. The survey found that the COVID-19 lockdown had varied impact on exercise adherence of the older adults: 43% of the participants exercised less during the lockdown than pre-lockdown, but 26% exercised more. Interestingly, among the participants approximately 80% still achieved the recommended physical activity level by the WHO during the lockdown. The focus groups revealed that exercise behaviours before the lockdown directly affected the behaviours during the lockdown. Participants’ recognition of the support from trustworthy people also influenced their motivation to perform exercises in an isolated environment. Remote exercise programs, such as digital and printed exercise materials, were found beneficial for the participants only when they came from the people the older adults trusted through their previous experience (i.e., the program). A sense of belonging to the exercise group was also essential for the participants to achieve self-managed exercise. It was concluded that older adults need connections to an exercise group and a trustworthy exercise instructor who could continuously support them to be physically active in isolated situations such as lockdowns, in addition to exercise knowledge and a better understanding of the benefits of exercise.

## Introduction

Normal had become abnormal, and the abnormal has become the new normal since the World Health Organization (WHO) first confirmed a new virus outbreak (COVID-19) in January 2020 [1]. This phenomenon has changed people’s daily lives worldwide, and Australia is no exception. Physical restrictions, including lockdowns, have had significant impacts on physical activities in Australia, ‘a sporting nation’ [2] from the time of the first COVID-19 active case identified in late January 2020 [3]. Approximately 20% of Australian children (aged 0–14 years) and adults (aged 15 years and over) who had participated in organised sports before the COVID-19 pandemic gave up the sports entirely by March 2021 [3]. Even in the state of Western Australia (WA), one of the relatively COVID-free locations, 30% of adults aged over 18 years were less engaged in physical activities (exercises and sports) during the COVID-19 period than before [4]. This reduction had severe impacts on older adults, especially on their physical and mental fitness [5].

According to the WHO’s physical activity guidelines [6], older adults should be engaged in a minimum of 30 minutes of moderate physical activity at least five days a week, preferably every day, to reduce the risk of developing chronic diseases, improve the immune system, mental health, and the quality of life. However, the 2017–2018 National Health Survey by the Australian Bureau of Statistics [7] reported that 69% of males and 75% of females aged 65 years and over did not achieve the recommended physical activity even before the pandemic. Thus, a considerable concern existed that the pandemic-related self-isolation/lockdowns would increase sedentary lifestyles in older adults, which would cause various negative impacts on their physical and mental health. In addition, this could lead to an increase in the number of health-related national expenditures [8].

A recent systematic literature review paper [9] found that lockdowns decreased physical activity or increased sedentary time among older adults; however, pre-lockdown habits affected those changes, and practical lifestyle interventions minimised the negative impacts caused by the COVID-19 pandemic. Lee and colleagues [10] also reported that a 2-month lockdown in Singapore increased depressive symptoms and decreased physical activity among older people, but more targeted interventions would mitigate the negative impacts of the future lockdown. There has been an increase in studies related to understanding older adults’ physical activity experiences and behaviours during the COVID-19 pandemic [e.g., 11–13]. For instance, public health guidance published in 2021 by the National Institute for Health and Care Excellence (NICE) in the UK suggests that digital and mobile health interventions could be an effective health tool for supporting community-dwelling older people physically active [14] or videos can be an effective tool for older adults to cope with stress and stay physically active [15]. In addition, Christensen and colleagues [16] have emphasised the universal promotion of physical activity in response to the significant physical activity declines due to the COVID-19 pandemic across whole societies around the world, particularly in older adults, to stop the further increase in inactive lifestyles.

Some theories exist concerning exercise behaviour, such as the health belief model [17], protection motivation theory [18], theory of reasoned action [19], theory of planned behaviour [20], or social cognitive theory [21]. Sirur and colleagues [22] advocated that theory-based exercise interventions were critical to achieving exercise adherence within a targeted population. However, they also insisted that one theory alone could not explain definite determinants to predict people’s physical activity behaviour. They stated that as the concepts of self-efficacy and outcome expectations were common factors across the prominent theories, it should be crucial to identify the mediators of the self-efficacy and outcome expectations, depending on the targeted population. For instance, Collado-Mateo and colleagues [23] identified 14 factors to increase adherence to physical exercises in older adults, emphasising that exercise adherence was affected by multiple factors; thus, intervention should be controlled and modified by those factors. Under those circumstances, it is significantly vital to deepen our understanding of the background of the older adults’ exercise experiences and behaviours before, during, and after the COVID-19 pandemic.

In order to seek strategies to keep older adults physically active during self-isolated situations like lockdowns in the future, it is inevitable to investigate the actual impact of a COVID-19 lockdown on the physical activity behaviours of older adults. Western Australia had the first one-month lockdown in April 2020, although the number of infected community cases was small. The authors have been running community-based exercise programs for older adults in WA’s metropolitan region, and the lockdown forced us to suspend the program. However, unlike the reported high 20% sports termination ratio after the COVID-19 pandemic in Australia [3] most of our program participants returned to the program after the lockdown. This provided us with a significant opportunity to investigate the impact of the COVID-19 lockdowns on the physical activities of the older adults who had attended a community-based exercise program before the lockdown and probe the rational background of their exercise behaviours. This also made us better understand what factors would mediate the self-efficacy and outcome expectations to exercise regularly for community-dwelling older adults in an isolated environment, including how pre-lockdown exercise experience and knowledge would affect older adults when they face the necessity of self-managed exercises. The findings from the present study are expected to help develop strategies for older adults to be physically active in the post-pandemic world where other lockdowns or similar isolated environments are possible.

## Methods

### Ethics statements

The Edith Cowan University Human Research Ethics Committee granted the study’s ethical approval (REMS No: 2020-01766-NOSAKA). The authors obtained written formal consent to participate in this study from individual participants.

### Study design

A two-phase mixed methods under an embedded design [24,25] was employed to examine exercise-related perceptions and experiences of older adults in WA in relation to the COVI-19 pandemic and lockdowns. In phase one, quantitative and open-ended data were collected using a self-administered questionnaire to find older adults’ exercise experiences and behaviours before and during the WA’s first a-month lockdown in April 2020. In phase two, qualitative data were obtained through focus group interviews to probe the findings of phase 1 and clarify critical elements to keep older adults physically active in future lockdown or similar isolated situations. The present study focused on how the COVID-19 lockdown affected exercises and physical activities in older adults who had previously participated in a community-based exercise program to investigate whether the exercise program impacted their ability and exercise knowledge to exercise alone during the lockdown. Therefore, participants were recruited using a purposeful sampling method targeting older adults (60 years and over) who participated in the program before March 2020 and came back to the program after the lockdown in July 2020. The program consists of 2.5-hour weekly session for eight weeks; one hour of physical exercise and one hour of cognitive health session with a morning tea in-between. Four programs per year have been run, and each program has attracted 20–40 participants. The lockdown in this study included travel and gathering restrictions: the older adults were required to stay home except for shopping for essentials, medical needs, up to one hour of exercise, or work, always wear a mask outside and have no visitor [26]. The participants who were in the program before the WA’s first lockdown in April 2020 are described as ‘pre-members’, and those who joined the program after the lockdown as ‘post-members’ in this study.

In the present study, the exercise experience during the lockdown was measured, based on frequency distributions of self-reported exercise experience: less exercise than before the lockdown, more exercise than before the lockdown, or no change. Exercise patterns were investigated using self-reported exercise time per week and exercise types performed during the lockdown (see S1 Text for the survey questions). This followed the evaluation approach that the public research institutions, including the Australian Sports Commission [3] and the Epidemiology Branch of the Western Australian Government [4]. In the modern era, no one had experienced a pandemic until the COVID-19; therefore, to the best of our knowledge, no previous studies evaluated exercise experiences or exercise patterns in older adults under pandemic-enforced lockdowns, thus, we think that this approach was appropriate. Open-ended responses were gathered in the survey to outline the rationale of the experience and patterns, and we probed the questions in the focus groups based on the survey (see S2 Text for the focus group semi-structural questions).

### Survey study

Fifty participants in a community-based exercise program for older adults in a metropolitan area of WA were invited to the survey verbally, via email or by phone through the program director after the WA’s first lockdown (July 2020). Forty-eight participants answered the questionnaire, and written informed consent was obtained from each participant (96% response rate). Among 48 respondents (32 females and 16 males), 32 were pre-members, and 16 were post-members. No significant (p>0.05) differences in gender component between groups, and age (pre-member group: 66–95, 75.5 ± 6.6 y, post-member group: 60–88, 71.4 ± 8.1 y) were evident.

A questionnaire was developed based on the best practices for survey research [27]. The final questionnaire for pre-members included eight closed and seven open-ended questions, and six closed and six open-ended questions were given to the post-members (see S1 Text for the survey questions). Printed self-administered survey questionnaires were distributed to the program participants who consented to participate in the survey at the beginning of the two programs after the lockdown on the 15th and 16th of July 2020. The respondents completed and returned the questionnaires to the program director by the end of each program.

Data were analysed using Excel spreadsheets for open-ended questions and IBM SPSS Statistics 27 [28] for closed-ended questions. Coding analysis classified the open-ended data and found patterns. Descriptive statistics captured the respondents’ characteristics, exercise experience, and exercise patterns.

### Focus groups

Active program members (who started the program before the lockdown and completed the survey) were invited to focus groups by email and in person through the program director. As a result, 26 members (20 females and 6 males) participated in one of three focus groups, 22 were pre-members who completed the survey, and the rest of the four were post-members who started the program after the lockdown. A written informed consent was obtained from each participant. One academic, who had never been involved in the program but had extensive focus group experience, facilitated the focus groups, which helped the focus groups minimise bias from existing relationships between facilitators and participants and maximise disclosure. One of the investigators took notes of the focus group date and time, participant numbers, questions asked by the facilitator, and key themes that emerged from the three discussions. The interviews were held on the 17th, 18th, and 31st of March 2021 in the facility’s meeting room, where the exercise program runs. Focus groups 1 and 2 had seven participants each (Group1: 5 females and 2 males: mean age = 76.9, Group2: 5 females and 2 males: mean age = 78.3). There was one post-member in Focus group 1 and two in Focus group 2. All twelve participants in Focus group 3 were pre-members, including 10 females and two males (mean age = 74.6). Each interview lasted about one hour, adhering to the WA Government’s COVID-19 rules (venue attendance registration, 2-square meter social distancing, and hand sanitiser application) and keeping all venue windows open. It should be noted that there was no positive case in the WA communities when the focus groups were conducted.

The data collected was digitally recorded using semi-structured focus group interviews. The semi-structured questions were developed to prove the results in the quantitative phase, based on the findings of phase one (see S2 Text for the focus group questions). After focus group 1, the focus group notes taken by the investigator and her feedback were used to assess the suitability of the semi-structured questions. That process confirmed the questions’ suitability; therefore, the initial semi-structured questions were used throughout the three focus groups. All researchers discussed and agreed on data saturation after the third focus group, referring to the focus group notes and feedback from the note-taker, and decided on the termination of other focus groups. The recorded data was transcribed verbatim by one investigator of this research with the assistance of the YouTube auto-captions. The notes taken were not directly included in the data analysed and reported in this manuscript; however, they were referred to in the data analyses. The transcribed data were analysed using Nvivo12 [29] in a causal explanation analysis [27,30–32]. Two investigators independently coded the data to categorise that into different events and behavioural/attitudinal patterns. The coded and categorised data were compared between the two investigators. A third investigator undertook a further review if a consensus was not reached. This procedure continued until all the investigators reached an agreement on the coding and categorisation, followed by the three investigators independently producing a causal map based on the coded/categorised data. They subsequently compared the maps and reanalysed them when their consensus was not made. The steps continued until the investigators agreed with the causal networks of the influences on exercise behaviours pre, during, and after the COVID-19 lockdown.

## Results

### Survey study

#### Exercise experience during the lockdown

Exercise experience of participants in the study following the lockdown phenomenon in April 2020 was assessed on a self-reported exercise experience, indicating either ‘less exercise than before the lockdown’, ‘more exercise than before the lockdown’, or ‘no change’. As shown in Table 1, 20 people (42.6%) exercised less, 12 people (25.5%) exercised more during the lockdown than the period before the lockdown, and 15 people (31.9%) indicated no change in their physical exercise before and during the lockdown period. There was no significant difference between the pre-members and post-members in their physical exercise experience during the lockdown (*p* = 0.923). These results indicated that the COVID-19 lockdown affected the exercise amount in approximately 68% of the participants either negatively or positively.

**Table 1:** Self-reported exercise experience during the first lockdown in April 2020 with Demographics of Questionnaire Respondents in Perth, Western Australia.

Variable	Total n (%)	Pre-member n (%)	Post-member n (%)
**Exercise amount**			
Less than before lockdown	20 (42.6)	14 (45.2)	6 (37.5)
More than before lockdown	12 (25.5)	8 (25.8)	4 (25.0)
As much as before lockdown	15 (31.9)	9 (29.0)	6 (37.5)
**Exercise time (min/week)**			
≤ 30	1 (2.2)	1 (3.2)	0 (0.0)
31–60	2 (4.4)	1 (3.2)	1 (7.1)
60–149	6 (13.3)	4 (12.9)	2 (14.3)
≥ 150	36 (80.0)	25 (80.6)	11 (78.6)
**Numbers of different exercises**			
1	5 (11.1)	2 (6.5)	3 (11.1)
2	15 (33.3)	6 (19.4)	9 (64.3)
3	10 (22.2)	8 (25.8)	2 (14.3)
≥ 4	15 (33.4)	15 (48.3)	0 (0.0)
**Three main exercise types**			
Exercise 1	Walking 42 (87.5)	Walking 28 (87.5)	Walking 14 (87.5)
Exercise 2	Eccentric exercise 24 (50.0)	Eccentric exercise 23 (71.9)	Yoga/Tai-Chi/Stretching 3 (18.8)
Exercise 3	Chair exercise 19 (39.6)	Chair exercise 17 (53.1)	Pilates 3 (18.8)

#### Exercise patterns during the lockdown

Exercise patterns were assessed from the exercise amount during the lockdown, the number of exercises performed, and the performed exercises. As shown in Table 1, while 42.6% of the participants reported that the COVID-19 lockdown reduced their exercise amount compared to the period before the lockdown, about 80% still achieved the recommended physical activity amounts (i.e., 150 min/week). This achievement ratio was much higher than that the national average reported by the Australian Bureau of Statistics [7]. It appears that the participants of the program were motivated to exercise regularly. It is possible that they attended the program because they had been already motivated well, or the exercise program made them exercise regularly.

The number of exercises performed during the lockdown showed a remarkable difference between the two groups. The pre-member participants reported that they performed more than four different exercises most during the lockdown; however, the post-member participants indicated that they performed two different exercises most. Walking was the most common for both groups (87.5%) during the lockdown, but high proportions of the pre-members also performed “eccentric exercise” (71.9%) and “chair exercise” (53.1%) that had been instructed in the program that they had attended before the pandemic. These showed that people in the pre-member group performed the exercises that they had experienced in the program before the lockdown. In other words, they applied their exercise experience and knowledge acquired through the program to their self-managed exercise during the lockdown.

In addition to the program, printed materials showing the eccentric and chair exercises with pictures to be performed at home were sent to the participants after entering the lockdown. The materials seemed to encourage the pre-members to do the eccentric exercises as well as the chair exercises during the lockdown. The results suggested that the previous attendance in the community-based exercise program helped the participants perform various exercises in the lockdown.

#### Encouragement factors to exercise

Thirty-three people provided open-ended answers about what encouraged them to exercise in the lockdown. Those included "self-determination" (27%), "social interaction" (23%), "resources" (23%), followed by "reminder" and "fun" (6% each). Notable differences between the pre-and post-members were that the pre-members’ descriptions categorised as “self-determination” described more precisely what exercise routine settings and how to stick to the routines to encourage exercise in isolations. On the contrary, the post-members only focused on general physical movements. The participants in both groups stated that exercising with a social interaction element, such as exercising with their family and friends, could encourage them to achieve regular exercise even in isolated situations. The materials to explain exercises, and resources, including exercise guides sent from the program organisers (e.g., exercise videos and handouts), were also described as essential motivators for regular self-exercise in isolation. Pre-members also stated that reminders from the program helped them keep exercising during the lockdown.

#### Remote exercise programs/materials

The program director emailed the links to three home-based exercise videos and printed guides as attachments to all the program participants immediately after the announcement of the first lockdown. The pre-members evaluated the materials using five answering options (1 = never used, 2 = slightly helpful, 3 = moderately helpful, 4 = very helpful, and 5 = extremely helpful). Sixteen respondents (53.3%) reported that the exercise guides were helpful to do exercises during the lockdown. The participants also provided descriptions of the main reasons for their judgements. The descriptions were coded and categorised. The main reasons for the helpfulness were ‘exercise prompts’ (76%) and ‘easiness to follow’ (18%). Some respondents put the printed guides on a refrigerator to be seen anytime they were in the kitchen to motivate them to exercise. ‘Easiness to follow’ was also a key element determining the helpfulness level of the remote exercise guides. The respondents found the remote exercise guides unhelpful to do exercise during the lockdown when they had ‘no motivation to exercise’ (47%) or ‘having a self-exercise menu’ (13%) and saw the guides ‘need a sense of companionship’ (13%). It is important to note that 27% of the respondents reported that they had unavailability to access the videos or printed guides due to unfamiliarity with technology or no home printers.

### Focus groups

**Fig 1:**
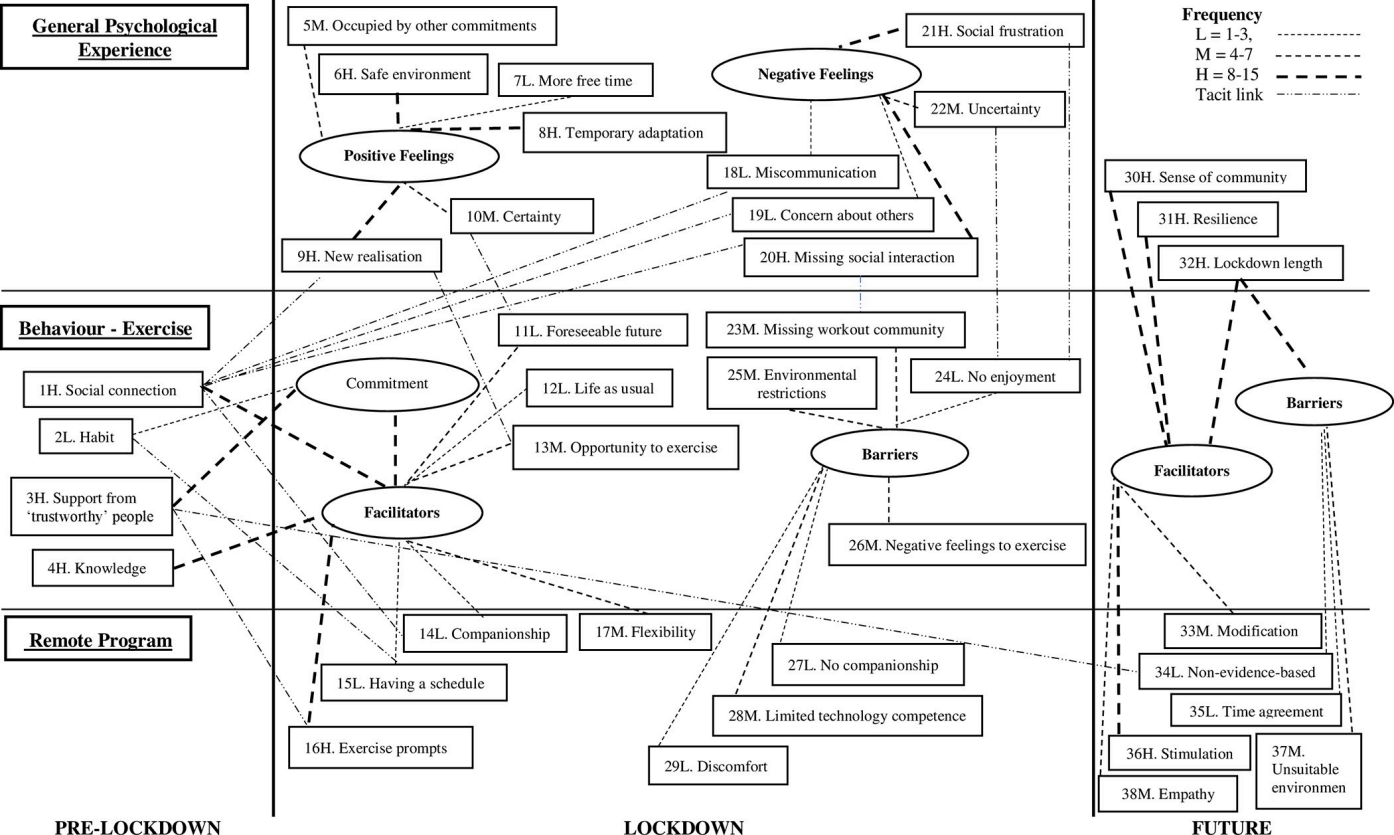
Chronological causal network: The influences on exercise behaviours and psychological responses before, during, and after the COVID-19 lockdown.

Qualitative data analyses of focus group sessions identified 38 influential elements of exercise behaviours and psychological responses during pre-lockdown, lockdown, and post-lockdown and their causal relationships, as shown in Fig 1. S1 Table summarises the coding framework, including code names, frequency, descriptions, and citations of the 38 elements.

#### Pre-and during lockdown exercise behaviours

As shown in Fig 1, tacit causal relationships were found between pre-lockdown exercise behaviours and general psychological experience during the lockdown. Social connections built before the lockdown tacitly but positively affected the general psychological experience of the participants. They re-realised the importance of social connection and found that even the connection they had not realised before the lockdown brightened up their mood. It was also found that exercise behaviours before the lockdown directly affected the behaviours during the lockdown. Social connections built before the lockdown and exercise-related knowledge obtained before the lockdown contributed to older adults’ independent exercise, working as exercise facilitators. Both the participants’ exercise habits and recognition of the exercise support from trustworthy people before the lockdown formed their commitment to exercise, and that commitment worked as one of the exercise facilitators during the lockdown. In addition, the general psychological experience tacitly affected the exercise behaviours during the lockdown. When the participants felt certainty, they were able to foresee what would happen next, which encouraged them to engage in exercise. The participants’ new realisation of the exercise importance, new exercise opportunities, and additional time for exercise also tacitly led them to see the lockdown as an opportunity to exercise.

On the contrary, the participants’ uncertainty tacitly caused anxiety–no enjoyment, which prevented them from exercising. Social frustration—annoyance by those who did not follow the COVID-19-related restrictions or those who complained about the restrictions also tacitly caused no enjoyment within the participants. As a result, they tended to be discouraged from exercising during the lockdown due to negative feelings towards the environment surrounding them.

#### Remote programs/materials and companionships

When the participants found the remote programs with companionships, they seemed motivated to work out. The flexibility of the programs also played a moderately important role in that. However, when the participants felt no companionship with the remote programs or found doing the remote programs discomfort, they were not motivated to exercise. In addition, it also became clear that the limited technological competence of the participants hindered them from engaging in exercise. In other words, older adults feel as if they are rejected when they cannot access remote programs or materials due to their technological competence. This type of feeling could discourage them from exercising. The results reported here may sound too obvious; however, the factors mentioned in this section require more attention to including them in a remote program or material when the program or material is developed. Older adults want to feel companionship from program/material contents and even from how they are delivered.

#### Sense of community, resilience, and remote program/material quality

The participants’ general sense of community, resilience towards lockdowns, or short lockdown period were also vital elements in facilitating exercise in the future. At the same time, a long lockdown period would hinder them from exercising. It was suggested that effective remote programs should be stimulative, flexible, and empathetic in the future. If the participants regard the remote programs as exercise stimulation, it will be highly possible to exercise; however, if the participants consider the remote programs not suitable to their environment, time, or non-evidence-based, they will not be engaged in an exercise in the future. The participants also emphasised the importance of variations of the remote programs and materials. No matter how good the programs and materials are, they need to be advanced and refined continually to stimulate older adults.

#### Effective remote programs and materials

Apart from the analysis reported above, thematic analysis was also conducted to identify the critical features of an effective remote material/program from the participants’ perspective. The features were found in three dimensions: preferred program features, delivery methods, and distribution frequency. The participants favoured a remote material/program that could extend their exercise community even during a lockdown. Several participants made copies of the exercise materials sent to them by the instructors during the lockdown and handed them to their family members, friends, and neighbours. The materials became a tool for older adults to connect or expand their community. Some dropped the copy off in their neighbours’ letterbox, and some others forwarded the electronic materials to their friends and families overseas. They also preferred a remote material/program with options, e.g., programs to conduct both outside and inside or small group programs outside in line with the required social distance and group gathering numbers. For them, even a remote material/program should contain a sense of community. They also considered the materials/programs something that can reinforce their willingness to exercise as one participant stated that “*I think it is important to have things to look forward to*”.

The participants suggested three delivery methods: 1) electric, such as emails, online meeting applications, online on-demand, and mobile texts, 2) in-person delivery, such as university students dropping a hardcopy material in a letterbox and having a short conversation with the participants through the front door, 3) posted hardcopy materials. Interestingly, preferred delivery methods appear to depend on the participants’ lifestyles. Some worried about paper consumption and posting costs for hardcopy materials, and others disliked electric methods concerning internet fraud. However, all participants agreed that the materials/programs should be delivered via multiple delivery channels.

The most preferred distribution frequency was when information was updated on the one hand; the second most preferred frequency was once a week on the other hand. One of the participants supported the remote material/program distribution once a week as the once-a-week material/program delivery means for her a social connection and fun of waiting to happen.

## Discussion

We investigated the impact of the COVID-19 lockdown on community-dwelling older adults who had attended a community-based exercise program before the lockdown to seek strategies to keep them active during other self-isolated situations. The study collected quantitative data on self-reported exercise experience, exercise amount (minutes per week), the number of exercises performed, and the exercise activities during the COVID-19 lockdown of the older adults and qualitative data through three focus groups to probe the quantitative findings and achieve the objective.

The quantitative data analyses found that the COVID-19 lockdown reduced the exercise amount in the majority of the older adults, but many maintained the recommended amount (150 minutes per week). These findings suggested that the participants adhered to exercise at the recommended level despite decreasing their exercise amount. The focus group discussions explored the rationale for the adherence behaviour. Exercise knowledge, reminders, and encouragement from someone trusted and expert, and a sense of social interaction are essential for older adults to remain physically active and minimise sedentary behaviours. The present study also revealed that the older adults who had attended an organised exercise program before the lockdown performed more variety of exercises independently than those who had not.

The focus group participants reported that what they learnt in the program helped them become confident in their exercise knowledge and better understood the negative impacts of a sedentary lifestyle on their physical and mental health, which encouraged their intention to keep exercising during the lockdown. Knowledge and confidence are self-efficacy, and understanding is outcome expectations. As suggested by the prominent theoretical models mentioned in the introduction, knowledge and better understanding increased the intention to exercise in the participants. However, as indicated by Sirur et al. [22] and other researchers [33,34], they were still insufficient to maintain older adults physically active; intentions should be transformed into behaviours when they work with other factors. The participants admitted that they needed cues to transfer their exercise intentions to actual performance, although they knew how to exercise and why they should keep exercising. The participants recognised the gap and saw that the exercise materials (e.g., handouts, videos) were one of the cues to help them into actual exercise behaviour. However, at the same time, the focus groups also revealed that older adults did not necessarily accept any materials, and they were incredibly selective and even suspicious of such material. When such materials do not meet their preferences and win their credibility, they will never use them. Furthermore, they do not accept anything if treated as ‘weak’ or ‘ignorant’ people; they always desire to deepen their knowledge and expand their skills like young people.

Additionally, the participants emphasised that the reliability of the material resources and a sense of social connection in the materials were also critical factors for achieving material acceptance. They particularly paid attention to who prepared the materials. If someone they already trusted qualified them, they counted the information and were willing to use it in an isolated environment. It became clear that building such rapport with the resource after being in an isolated environment is challenging for older adults. Therefore, it is inevitable for them, or those who would support them in the remote environment, to have a trusted relationship with the resource providers/communicators beforehand. Community-based programs could be one of the best ways to achieve this. Timely access or information sharing in the sense of community from program organisers also plays a vital role in keeping older adults physically active in an isolated environment. Older adults are motivated to exercise when they feel that they are not emotionally isolated and know someone close to them is also in the same situation and try to keep exercising. Hystad and Carpiano [35] have also confirmed a strong positive relationship between a sense of community belonging and health behaviour in Canadians. Therefore, setting up the most accessible measures to deliver the information or contact older adults is crucial.

The focus groups additionally revealed that older adults preferred multiple selections of material formats. Some were familiar with the internet and new devices; others stuck to traditional (e.g., more familiar) forms, including printed materials and landlines. Although technologies are advancing and digital information has become standard for the general population, it should not be granted that older adults are also a part of that. The Office of the eSafety Commissioner, the Australian Government [36], reported a strong relationship between age and digital literacy; the older, the more digitally disengaged. Therefore, older adults still require various material formats and delivery methods to cover the wider older population. The focus groups proposed email, digital attachment, link sharing, online meeting tools, landlines, and postal service for hard copies. It also became clear that the older adults confirmed connecting to others by receiving the materials in an isolated environment. They do not necessarily have to receive such materials daily or weekly; however, they must know in advance that they will be updated or accessed at least once a month to ensure they will not be forgotten.

Based on the findings mentioned above, we propose a new initiative strategy called “BE ACTIVE” to facilitate older adults to exercise independently even when their living environments suddenly change, or they are forced to be in an isolated environment: the strategy is encouraging older adults to 1) belong to the reliable exercise community and build a good relationship/friendship with other community members/supporters, 2) acquire exercise knowledge and skills from experts, 3) seek assistance from trustworthy people, 4) be informed and updated regularly, 5) ensure they connect with others constantly, 6) be confident in their ability to perform an exercise, 7) inspire others to get into exercise, enjoy various types of exercise, and 8) build up resilience against an isolated environment.

### Limitations of the study

First, when generalising the findings of this study, modifications will be required, depending on cultures, regions, or countries older adults live in, as the study was based on the WA community-dwelling older adults. In addition, WA lockdowns to date have been softer than those in other states and countries. Hence, there would be other vital factors for physical activity in older adults in a restricted environment that this study has missed. Second, the effectiveness of the proposed initiative is unclear for sedentary older adults as this study did not deal with motivations for sedentary older adults to start exercising. Additional study with the inactive population is required for applying the initiative to the larger population. Gender bias in the collected data could affect the findings of this study. Although the female orientation in the participants of this study is typical within community-based programs, more male data will need to be collected and analysed to achieve more active older adults generally. Finally, other approaches could be possible to investigate exercise experience, such as weighted aggregate scoring of outcome variables of exercise experience, although the reliability and validity of the scoring under isolated conditions like the COVID-19 lockdown require justification.

## Conclusions

This study found the COVID-19 lockdown affected exercise amount among older adults who attended a community-based exercise program. However, the present study identified essential factors that would make older adults stay physically active even when they were forced to be isolated where their exercise opportunities are restricted. Older adults need to know about exercise and the benefits of exercise, have companionship connections with a community-based exercise group, have trustworthy exercise instructors and programmers, and receive updated exercise-related information to stay physically active in an isolated environment. Future research should investigate the effectiveness of the essential factors and conditions identified in the present study for changing older adults’ physical activity behaviour. Suppose the effectiveness or other additional factors are confirmed, older adults will accept the new normal and stay active even when provided physical activity opportunities are suddenly restricted or they cannot attend a community-based exercise program. Physical activity behaviours cannot be changed suddenly; thus, what older adults are currently doing determines their behaviours in situations that could restrict their physical activities, such as lockdowns. The convergence of the COVID-19 pandemic is approaching, and we should urgently promote exercise for older adults for a healthier future.

## Supporting information

S1 Table Coding framework: Pre-lockdown, during lockdown, and future. (DOCX)

S1 Data Older adults exercise data variables. (XLSX)

S1 Text Survey questions. (DOCX)

S2 Text Focus group interview: Questions. (DOCX)
