# Supplementary material for: Impact of COVID-19 lockdown on physical activity behaviours of older adults who participated in a community-based exercise program prior to the lockdown
Source: PLOS Glob Public Health. 2022 Nov 11;2(11):e0001217. doi: 10.1371/journal.pgph.0001217 (PMC10022279; doi:10.1371/journal.pgph.0001217)
Supplement: S1 Table — (DOCX) [file pgph.0001217.s001.docx]

**S1 Table: Coding Framework: Pre-lockdown, during Lockdown, and Future**

| ***When & Category*** | ***Code*** | ***Frequency**** | ***Description*** | ***Citation***** |
| --- | --- | --- | --- | --- |
| **Pre-lockdown**  **Behaviour-Exercise** | 1. Social connection | H | People feel connected socially. | That’s a community group where people all want to do the same thing, they could have liked interest. (FG1-M2) |
|  | 2. Habit | L | Exercise is a habit. | You’re actually doing stuff because I did it every day, every day, you know. (FG3-F10) |
|  | 3. Support from ‘trustworthy’ people | H | Information/support is provided by trustworthy people. | I think what Ken’s uh exercises and the way I had been doing them for about five years actually and I just immediately thought that’s what I’ll be doing. (FG1-F1) |
|  | 4. Knowledge | H | People have knowledge on exercise. | What I find very attractive about Ken’s exercises is the fact that they’re so efficient. You can you know that all that research you did with regard to how efficient they are how beneficial they are compared to more conventional exercising. (FG2-M1) |
| **During Lockdown**  **General psychological experience** | 5. Occupied by other commitments | M | State where other commitments occupy people. | COVID didn’t play such a big role really because I was just so preoccupied (FG3-F5) |
|  | 6. Safe environment | H | State where people feel safe. | I think we were very well led and directed whether you liked Mark McGowan or not sure I think he did a great job in keeping people of you know like it was these are the rules and look after each other and it was reinforced all the time wasn’t it? (FG3-F2) |
|  | 7. More free time | L | State where people have more free time. | I was so relieved because I didn’t have to go out to any appointments. (FG1-F2) |
|  | 8. Temporary adaptation | H | State where people feel they are adapted to abnormality. | I thought people were wonderful here that they adjusted to it. (FG1-F1) |
|  | 9. New realisation | H | State where people realise new things. | I was very impressed that the very first time you know it came up that you know there was this infection then look after your neighbours sort of thing and we had a piece of fruit cake and a lovely card with it with the mobile phone numbers of a couple of the young ladies immediately with us. (FG3-F1) |
|  | 10. Certainty | M | State where people feel certainty. | We all knew it was only going to be a week well you hoped it was um a time out. (FG1-M1)  I think it’s also a bit easier this period of time because we knew vaccines were coming off. (FG1-M2) |
|  | 18. Miscommunication | L | State where people find miscommunication with others. | They (my friends and family overseas) keep thinking are you not, are you this, and that, all they can’t understand. I am not in that, and you don’t really know what I am like, and they don’t understand that. (FG2-F2) |
|  | 19. Concern about others | L | State where people worry about others. | I felt concerned because all my non-direct family were in UK my sister was 80. (FG1-M2) |
|  | 20. Missing social interaction | H | State where people miss social interaction with others. | I felt also you know it was just something missing, people extending the day and you you know you had other things but it suddenly stopped (FG3-F1) |
|  | 21. Social frustration | H | State where people feel social frustration. | I was upset about the people, in greed. As an over 70 it probably felt discriminatory. (FG2-M1) |
|  | 22. Uncertainty | M | State where people feel uncertainty. | I immediately thought well I’m single living in a villa on my own. What’s what’s going you know how is it going to work. (FG2-M1) |
| **During Lockdown**  **Behaviour-Exercise** | 11. Foreseeable future | L | People find the future foreseeable. | I think it’s also a bit easier this period of time because we knew vaccines were coming off. This is a temporary thing until we but you know what the authority over by last May or June at the latest. (FG1-M2) |
|  | 12. Life as usual | L | People keep their life as usual. | I I found very little change in my lifestyle so that’ll shape I keep physically sharing shaped. (FG2-M1) |
|  | 13. Opportunity to exercise | M | People regard the lockdown as an opportunity to exercise. | So I think I was exercising more in that shutdown than I was before really because you have so much time. (FG-M1) |
|  | 23. Missing workout community | M | People miss the workout community. | if it was a little bit longer it might have felt like a community was taken away in some routine there. (FG3-F2) |
|  | 24. No enjoyment | L | People see exercise during the lockdown as unenjoyable. | Oh I just didn’t enjoy it (YouTube/Zoom exercises). FG2-F3) |
|  | 25. Environmental restrictions | M | People find environmental restrictions to exercise. | For me it was more frustrating because of having to wear masks. (FG2-F3)  I couldn’t do uh guided yoga with my yoga teacher who used to actually come to our house in the once a week in the morning. (FG3-F10) |
|  | 26. Negative feelings to exercise | M | People feel negative to exercise. | I’m pretty hopelessly unmotivated. (FG3-F4) |
| **During Lockdown**  **Remote Program** | 14. Companionship | L | People feel companionship in the remote program. | I think the the YouTubes are excellent. I think there’s only two of them and especially the one because that’s actually had some of the Stay Sharp people here yes this is one of the fastest ideas so that was good I mean because it relates back to people like of our age yeah. (FG1-M1) |
|  | 15. Having a schedule | L | People see the program as having a schedule. | One of the things with the Zoom was um you know at 9.30 we’ve got it started you know and then there was another one after that which I didn’t normally go to but I did that as well because there was that set times which was really important. (FG3-F5) |
|  | 16. Exercise prompts | H | People see the remote program as exercise prompts. | I used the poster. I like this I’ve got a picture on the kitchen cupboard, right there constantly staring in the face.(FG1-F2) |
|  | 17. Flexibility | M | People find flexibility in the program. | I like the YouTube videos. I like those very much because you could stop and start whenever you wanted yeah well yeah you can, you can start doing it and then when click the button when it moves on to the next exercise and you can do a few more of these before just start it off again. (FG2-M1) |
|  | 27. No companionship | L | People do not feel companionship in the remote program. | I got those who stick to this there. If you speak to them in Zoom, that’s the heck. That’s not the same. (FG2-F2) |
|  | 28. Limited technology competence | M | People have limited technology competence. | I don’t do technology, I don’t do Zoom or any of that so. (FG3-F10) |
|  | 29. Discomfort | L | People find the program out of their psychological and physical comfort zone. | It’s your comfort zone if you’re comfortable like with with Ken’s videos and I would have liked to have done more of it. (FG2-F1) |
| **Future**  **General Psychological Experience** | 30. Sense of community | H | State people feel a sense of community. | Yeah it’s more I think as much as possible face to face. Technology is a sort of… (FG1-M1)  I just know everybody that’s first name know where they lived you know what I’ve done but but at least you’ve got something in common that you like. (FG1-F1) |
|  | 31. Resilience | H | State people feel they have resilience. | I think there’s a good chance we’ll have another lockdown. We’ve got more ambition to do with it. (FG3-M3) |
|  | 32. Lockdown length | H | State people regard the lockdown as long or short. | when you’re in for a long longer long time then you’ve got to well maybe you’ve got to sit down and structure your exercising and think what am I going to do and what are the options. (FG2-F2)  Two weeks that was fine. (FG3-F3) |
| **Future**  **Remote Program** | 33. Modification | M | People see modifications in a remote program. | You know Ken and a group of people that want to do the exercise space to play yeah but you have other people who’d be quite content doing through zoom. (FG1-M2) |
|  | 34. Non-evidenced based | L | People see a remote program be non-evidence based. | This is not uh this is just a general um comment about some of my negative things um a lot of programs are produced by either um well-known people or young people telling you hahaha we must be happy or um you know stuff to do to keep us and it’s all to me it’s all artificial um you know it’s not you don’t behave like that every day like absolutely no. (FG2-F3) |
|  | 35. Time agreement | L | People see a remote program requiring time agreement to attend. | But, the time agreement, but not necessarily. (FG1-F2) |
|  | 36. Stimulation | H | People see a remote program as an exercise stimulation. | It’s just a very enjoyable program uh I’d come even if I was a world-class athlete and the novelty of the eccentric exercises yes it’s just worth it just for that. (FG2-M3) |
|  | 37. Unsuitable environment | M | People see a remote program set in an unsuitable environment. | It was hard to walk pass the narrow path on a bicycle with dogs. (FG2-F2) |
|  | 38. Empathy | M | People feel empathy in a remote program. | It (other programs) is all to me it’s all artificial um you know it’s not you don’t behave like that every day like absolutely no. (FG2-F3) |
| * L = 1-3 references, M = 4-7 references, H = 8-15 references  **For achieving confidentiality, each participant is coded using their group number, gender, and respondent number, e.g., FG1-F1 (Focus group 1, female respondent 1) | | | | |
