# Supplementary material for: Impact of COVID-19 lockdown on physical activity behaviours of older adults who participated in a community-based exercise program prior to the lockdown
Source: PLOS Glob Public Health. 2022 Nov 11;2(11):e0001217. doi: 10.1371/journal.pgph.0001217 (PMC10022279; doi:10.1371/journal.pgph.0001217)
Supplement: S1 Text — (DOCX) [file pgph.0001217.s003.docx]

**S1 Text: Survey Questions**

1. Name: __________________
2. Age: _______ years old
3. Gender: M / F
4. How many terms have you attended the program previously (please circle)?

One, Two, Three, Four, Five or more

1. What frequency per week and type of exercise did you regularly do before the coronavirus lockdown?
2. Please choose your exercising experience during the coronavirus lockdown. Circle the number you chose from the following.
   1. I exercised less than before the lockdown.
   2. I exercised more than before the lockdown.
   3. I exercised as much as before the lockdown.
3. What are the main reasons why you exercised less than/more than/as much as before the lockdown?
4. What exercise did you regularly do during the lockdown? Please choose all that apply.
   1. Eccentric exercise _________minutes ­­__________ times/ week
   2. Chair exercise _________minutes ­­__________ times/ week
   3. Walking _________minutes ­­__________ times/ week
   4. Yoga _________minutes ­­__________ times/ week
   5. Pilates _________minutes ­­__________ times/ week
   6. Cognitive exercise _________minutes ­­__________ times/ week
   7. Other: ­­­­­ _________minutes ­­__________ times/ week
5. Did the information and video clips of home-based exercises help you sustain regular exercise during the lockdown?
   - Yes

Why?_­­­ ­­­­­­­­­­­­­­­­­____________________________________________________________________________

- - No

Why?_­­­ ­­­­­­­­­­­­­­­­­____________________________________________________________________________

| Material | Never  used | Not at all helpful | Slightly helpful | Moderately helpful | Very helpful | Extremely helpful |
| --- | --- | --- | --- | --- | --- | --- |
| VIDEO 1: Five simple exercises seniors can do at home | 0 | 1 | 2 | 3 | 4 | 5 |
| VIDEO 2: Aerobic Exercises on a chair | 0 | 1 | 2 | 3 | 4 | 5 |
| VIDEO 3: Eccentric Exercises | 0 | 1 | 2 | 3 | 4 | 5 |
| HANDOUT 1: Eccentric exercise | 0 | 1 | 2 | 3 | 4 | 5 |
| HANDOUR 2:  On chair exercise | 0 | 1 | 2 | 3 | 4 | 5 |
| HANDOUT 3: Cognitive exercise | 0 | 1 | 2 | 3 | 4 | 5 |

1. Please tick the most appropriate box of each material provided to you below.

1. What are the positive results brought about by your exercise experience during the lockdown?
2. What are the negative results brought about by your exercise experience during the lockdown?
3. What are the hardest parts for you in the lockdown in general?
4. How would you have been encouraged to do exercise regularly during the lockdown?
5. What communication tools would you like to use in a similar condition to the lockdown? Circle all the numbers from the following that apply.

10-1. Fixed phone talk

10-2. Mobile phone talk

10-3. Digital message (e.g., Mobile text, Email, Messenger)

10-4. Online video communication (e.g., Facetime, Zoom, Teams)

10-5. Other

(please specify ______________________________________)
