# Supplementary material for: Impact of COVID-19 lockdown on physical activity behaviours of older adults who participated in a community-based exercise program prior to the lockdown
Source: PLOS Glob Public Health. 2022 Nov 11;2(11):e0001217. doi: 10.1371/journal.pgph.0001217 (PMC10022279; doi:10.1371/journal.pgph.0001217)
Supplement: S2 Text — (DOCX) [file pgph.0001217.s004.docx]

**S2 Text: Focus Group Interview: Questions**

1. Greeting by Chief Investigator (CI)

- Information sheet & Consent form - reconfirmation
- Audio-recording - reconfirmation

1. Introducing a facilitator by CI
2. Greeting by the facilitator

- Build rapport to get frank opinions
- Make sure that the participants are welcome to attend the interview eating
- Show the materials (videos & handouts)

Q1. Can you tell me about your exercise experience during the 1^st^ and 2^nd^ lockdown?

Probes:

- 1. Physical impact
  2. Mental impact
  3. Social impact
  4. Exercise motivation during the lockdown (Fitness, Health, Dementia prevention, Socialisation, Stay Sharp instructors/staff)

Q2. During the lockdown, which and how often did you use the materials?

Probes:

- 1. Why did you (didn’t you) use the materials you chose?
  2. What are the differences between the materials you used and didn’t use
  3. How could the materials be improved for your use?

Q3. When in a similar situation to the lockdown next time, what information/materials would you want to get to keep you exercising?

Probes:

- 1. Method: e.g., Phone, Live online stream, Online materials (video, sound etc.), Document, DVD. Explain & Why?
  2. Distribution method: e.g., Email, Snail mail, etc. Explain & Why?
  3. What and how much information would you need? Explain & Why?
  4. Is an element of socialisation of the materials vital for you to keep exercising?
  5. From whom would you like to get the information? Explain & Why?

Q4. Are you confident in keeping exercising when you are isolated, like during the lockdown? (self-efficacy)

Probes:

- 1. Would you think any materials, including the subjected materials, help keep exercising? Explain & Why?

Q5. Any other suggestions or opinions?

Close the interview

End
